# The exoribonuclease Polynucleotide Phosphorylase influences the virulence and stress responses of yersiniae and many other pathogens

**DOI:** 10.3389/fcimb.2013.00081

**Published:** 2013-11-19

**Authors:** Jason A. Rosenzweig, Ashok K. Chopra

**Affiliations:** ^1^Department of Biology, Center for Bionanotechnology and Environmental Research, Texas Southern UniversityHouston, TX, USA; ^2^Department of Environmental and Interdisciplinary Sciences, Texas Southern UniversityHouston, TX, USA; ^3^Department of Microbiology and Immunology, University of Texas Medical BranchGalveston, TX, USA; ^4^Sealy Center for Vaccine Development, University of Texas Medical BranchGalveston, TX, USA; ^5^Institute of Human Infections and Immunity, University of Texas Medical BranchGalveston, TX, USA; ^6^Galveston National Laboratory, University of Texas Medical BranchGalveston, TX, USA

**Keywords:** RNA decay, yersiniae, type three secretion system, host-cell induced stress response, degradosome, oxidative stress response, cold stress response

## Abstract

Microbes are incessantly challenged by both biotic and abiotic stressors threatening their existence. Therefore, bacterial pathogens must possess mechanisms to successfully subvert host immune defenses as well as overcome the stress associated with host-cell encounters. To achieve this, bacterial pathogens typically experience a genetic re-programming whereby anti-host/stress factors become expressed and eventually translated into effector proteins. In that vein, the bacterial host-cell induced stress-response is similar to any other abiotic stress to which bacteria respond by up-regulating specific stress-responsive genes. Following the stress encounter, bacteria must degrade unnecessary stress responsive transcripts through RNA decay mechanisms. The three pathogenic yersiniae (*Yersinia pestis*, *Y*. *pseudo-tuberculosis*, and *Y*. *enterocolitica*) are all psychrotropic bacteria capable of growth at 4°C; however, cold growth is dependent on the presence of an exoribonuclease, polynucleotide phosphorylase (PNPase). PNPase has also been implicated as a virulence factor in several notable pathogens including the salmonellae, *Helicobacter pylori*, and the yersiniae [where it typically influences the type three secretion system (TTSS)]. Further, PNPase has been shown to associate with ribonuclease E (endoribonuclease), RhlB (RNA helicase), and enolase (glycolytic enzyme) in several Gram-negative bacteria forming a large, multi-protein complex known as the RNA degradosome. This review will highlight studies demonstrating the influence of PNPase on the virulence potentials and stress responses of various bacterial pathogens as well as focusing on the degradosome-dependent and -independent roles played by PNPase in yersiniae stress responses.

## Introduction

Earlier, mRNA decay was considered a non-specific, rapid, and inevitable default pathway for all transcripts, regardless of their size and/or secondary structure (Deutscher and Li, 2001; Kushner, 2002). Presently, profound advances in our understanding of RNA decay/maturation processes and their regulation have been made (Deutscher and Li, 2001; Kushner, 2002; Silva et al., 2011). The majority of RNA decay studies have employed *Escherichia coli*, with limited (but a growing number of) studies performed in Gram-positive organisms (Condon and Bechhofer, 2011; Silva et al., 2011) including *Bacillus subtilis* (Even et al., 2005; Mäder et al., 2008; Bechhofer, 2011; Lehnik-Habrink et al., 2012), *Streptococcus pyogenes* (Barnett et al., 2007), *Streptococcus pneumoniae* (Domingues et al., 2009), and *Staphylococcus aureus* (Huntzinger et al., 2005; Boisset et al., 2007). Found in both Gram-positive and -negative organisms, ribonucleases are enzymes that degrade ribonucleotides. Endoribonucleases cleave RNA molecules within the transcript while exoribonucleases degrade RNA molecules in a 3′ to 5′ or 5′ to 3′ direction (Silva et al., 2011). Of all the Gram-positive organisms interrogated, *B. subtilis* has been most extensively studied regarding ribonucleases (Even et al., 2005; Mäder et al., 2008; Bechhofer, 2011; Lehnik-Habrink et al., 2012) and PNPase specifically (Oussenko et al., 2005; Lehnik-Habrink et al., 2012).

## RNase E and the degradosome

The 1061 amino-acid residue long ribonuclease E (RNase E) is the predominant endoribonuclease in *E. coli* and is essential for cell viability. It is absent in Gram-positive organisms which instead rely on endoribonucelase/exoribonuclease J (RNase J1/J2) (Condon, 2010; Silva et al., 2011). The first 500 amino-terminal RNase E residues include both the catalytic and S1 RNA-binding domains. The carboxy-terminus (residues 734–1060) serves as a scaffolding region upon which the various components of the multi-protein degradosome complex bind, including the exoribonuclease polynucleotide phosphorylase (PNPase), the RNA helicase RhlB or RhlE, and enolase (a glycolytic enzyme) (Carpousis et al., 1994; Vanzo et al., 1998; Khemici and Carpousis, 2004). Specialized degradosomes of differing composition (including the association of additional accessory proteins) as well as a eukaryotic degradosome-like machine (the exosome) have also been reported in *Saccharomyces cerevisiae* (Lawal et al., 2011 and references therein). More specifically, a degradosome containing RNase E, the exoribonuclease RNase R, and RhlE (an RNA DEAD box helicase) was identified in the psychrotrophic bacterium *Pseudomonas syringae* Lz4W (Purusharth et al., 2005). Additionally, a “cold-shock degradosome” was identified in *E. coli* consisting of the cold-inducible DEAD box helicase CsdA/DeaD, PNPase, and other degradosome components (Prud'homme-Généreux et al., 2004).

Despite physical evidence of degradosome assembly observed in a number of organisms, the precise physiological functions carried out by these large multiprotein complexes/hyperstructures have yet to be fully elucidated (Norris et al., 2012). Using bacterial two-hybrid analysis as well as co-immunoprecipitation techniques, we recently determined that a *Yersinia pseudotuberculosis* degradosome, comprised of RNase E, PNPase, and RhlB helicase associates (Henry et al., 2012), extending an earlier report of physical interaction between only PNPase and RNase E in the aforementioned organism (Yang et al., 2008).

### PNPase

Originally identified in *E. coli*, PNPase, encoded by the *pnp* gene, is an ~80 kDa homo-trimeric exoribonuclease that poorly degrades structured RNA molecules (Guarneros and Portier, 1991; Symmons et al., 2000). The *Yersinia pestis* bv. Antique's highly conserved PNPase (UniProt Entry: Q1C3L8) is a slightly smaller enzyme (~78 kDa) but shares 89% sequence similarity with that of its *E. coli* counterpart. Additionally, PNPase acts as a polymerase when the concentration of nucleotide di-phosphates is greater than inorganic phosphate, as shown in *E. coli* (Deutscher and Li, 2001). Historically, PNPase played a large part in determining the genetic code (Grunberg-Manago et al., 1955), winning Dr. Severo Ochoa the 1959 Nobel Prize in Physiology or Medicine. As mentioned above, PNPase is also a degradosome constituent in *E. coli* and *Y. pseudotuberculosis*, but exactly what percentage of PNPase's degradative effort is spent while associated with the degradosome has yet to be determined (Lawal et al., 2011). To date, the synthetic/polymerase activity of PNPase has not been demonstrated in the yersiniae. PNPase consists of two catalytic domains as well as both structurally conserved KH and S1 RNA-binding domains in its extreme carboxy-terminus.

### PNPase, non-coding sRNAs, and Hfq (RNA-binding protein)

With the discovery of functional, small non-coding RNA molecules (sRNAs) involved in regulating gene expression, the entire paradigm associated with “what constitutes a gene” was shifted. sRNAs became increasingly studied when they were found to influence bacterial physiology and even virulence (Gottesman et al., 2001; Shimoni et al., 2007; Repoila and Darfeuille, 2009; Silva et al., 2011; Storz et al., 2011). More specifically, in *E. coli*, PNPase was found to stabilize several regulatory sRNAs including: RyhB, SgrS, and CyaR. Further, RNase E appeared responsible for their increased turnover/decay in the absence of PNPase (De Lay and Gottessman, 2011). These findings strongly suggest that the degradosome assembly (which includes the presence of PNPase) is required for stabilizing some regulatory sRNAs in *E. coli*. Perhaps the degradosome is protecting virulence-regulating sRNAs in bacterial pathogens as well?

In *Y. pseudotuberculosis*, 150 sRNA species (Koo et al., 2011) have been identified, many of which are involved in *Yersinia* virulence (Koo and Lathem, 2012; Lathem, 2012). Upon proteome profiling, the SraG sRNA was found to regulate expression of 16 different genes as evidenced by differential protein expression in *Y. pseudotuberculosis*. Interestingly, SraG appeared to positively regulate levels of PNPase, one of the 16 differentially expressed proteins (Lu et al., 2012). These powerful sRNAs used for modulating gene expression complement other post-transcriptional regulating factors including: ribonucleases (e.g., PNPase), RNA-binding proteins, and thermal riboswitches (e.g., the low calcium response at 37°C) (Schiano and Lathem, 2012).

In many instances, Hfq (an RNA-binding protein) will bind to sRNAs either promoting their stabilization or degradation (Silva et al., 2011). In the case of *Yersinia pestis*, 31 unique sRNAs were discovered, and the majority of those required the presence of Hfq for their expression (Beauregard et al., 2013). A separate global analysis of sRNAs revealed as many as 43 species in *Y. pestis* (Qu et al., 2012). Further, of the two *Y. pestis* iron-responsive sRNA RyhB homologs, only RhyB1 required Hfq for stabilization while RyhB2′s stability was Hfq-independent (Deng et al., 2012). Further, PNPase has been shown to play a crucial role in the degradation of sRNAs that do not associate with Hfq (Andrade et al., 2012).

However, in *E. coli*, Hfq was shown to be necessary for PNPase-mediated degradation of the Cold shock Protein A encoding transcript (*cspA*) during cold shock (Hankins et al., 2010). Hfq also influences the virulence of a number of pathogenic bacteria (Chao and Vogel, 2010) including *Y. pestis* and *Y. pseudotuberculosis* (Geng et al., 2009; Schiano et al., 2010), while also influencing the growth of the latter (Bai et al., 2010). Perhaps Hfq is the link between yersiniae PNPase, sRNAs, and bacterial virulence?

### PNPase and cold shock/cold growth

Bacterial responses to low temperature stress are generally referred to as “cold shock.” PNPase confers cold growth capabilities to a number of bacteria (including several pathogens) including: *E. coli*, the yersiniae, *Salmonella enterica*, *Campylobacter jejuni*, *S. aureus*, and *B. subtilis* (Jones et al., 1987; Wang and Bechhofer, 1996; Goverde et al., 1998; Clements et al., 2002; Rosenzweig et al., 2005, 2007; Anderson and Dunman, 2009; Haddad et al., 2009, 2012). Considering that *C. jejuni* and several pathogenic yersiniae are contracted via the fecal oral route, PNPase could serve to promote bacterial persistence on processed meats that are shipped at refrigerated temperatures (thereby facilitating foodborne illness).

During the cold shock response in *E. coli*, cold inducible genes, including six cold shock protein genes (*csp*'s), become upregulated (Jones et al., 1987; Goldstein et al., 1990). Additionally, cold-inducible *pnp* and *rnr* genes, encoding PNPase and RNase R respectively, were also found to be cold-inducible in *E. coli* (Jones et al., 1987; Cairrão et al., 2003). Interestingly, RNase R was required for specifically degrading *ompA* transcript (encoding outer membrane protein A—OmpA) during stationary phase growth of *E. coli* (Andrade et al., 2006), strongly suggesting that RNase R is not only cold-inducible but also growth-phase inducible.

During acclimation, immediately following cold-shock, PNPase is required for degradation of unnecessary *csp* (that likely trap ribosomes) and other cold-inducible transcripts in both *E. coli* and *Yersinia enterocolitica* (Goverde et al., 1998; Neuhaus et al., 2000; Yamanaka and Inouye, 2001; Polissi et al., 2003). More specifically, in *Y. enterocolitica* PNPase, together with RNase E, influences cold growth adaptation through its recognizing and cleaving AGUAA motifs (termed cold-shock-cut boxes) in *csp* transcripts (Neuhaus et al., 2003). Furthermore, the ability of PNPase to promote cold growth in the yersiniae absolutely required the catalytic activity of PNPase and to a lesser extent its RNA-binding domains, the S1 RNA-binding domain more than the KH RNA-binding domain (Rosenzweig et al., 2005). Surprisingly, we found that PNPase was not required for chemical stress responses associated with antibiotic exposures that target protein translational machinery, the cellular membrane, and the yersiniae cell wall (Henry et al., 2012), indicating that PNPase might not be a global stress regulator but rather a regulator of specific encountered stresses in yersiniae, like the cold temperature stress.

### PNPase and bacterial virulence

PNPase influences the virulence of number of human pathogens as well as one animal pathogen. In the Gram-negative *Dichelobacter nodosus* (the etiological agent of foot root in sheep), PNPase was found to negatively regulate virulence/twitching motility, primarily through its S1 RNA-binding domain (Palanisamy et al., 2010). Whether animal or human pathogens, when Gram-negative pathogens, including the yersiniae and salmonellae, are confronted by threatening innate immune cells, a rapid genetic re-programming occurs resulting in anti-host type three secretion system (TTSS) genes becoming upregulated (Cornelis and Van Gijsegem, 2000; Cornelis, 2002; Viboud and Bliska, 2005). It has now been firmly established that a number of ribonucleases (e.g., PNPase, RNase R, RNase E, RNase III) influence bacterial virulence in a wide range of Gram-negative and -positive pathogens (Anderson and Dunman, 2009; Lawal et al., 2011; Jester et al., 2012; Matos et al., 2012). Specifically, PNPase was required for the optimal virulence of several bacterial human pathogens (Table 1), in several instances by targeting TTSS targets.

**Table 1:** **List of PNPase influences on various bacterial pathogens**.

**Organism**	**PNPase role**	**References**
*Salmonella enterica*	Promotes cold growth	Clements et al., 2002
	Promotes acute infection and virulence in murine infections	Clements et al., 2002
	Suppresses *Salmonella* plasmid virulence genes	Ygberg et al., 2006
*Yersinia enterocolitica*	Promotes cold growth	Goverde et al., 1998
*Yersinia pseudotuberculosis*	Promotes cold growth	Rosenzweig et al., 2005
	Promotes virulence in murine and cell culture infections	Rosenzweig et al., 2007
*Yersinia pestis*	Promotes virulence in cell culture infection and optimal TSSS function	Rosenzweig et al., 2005
	Promotes virulence in Swiss Webster murine IP infections	Lawal et al., 2011
*Dichelobacter nodosus*	Suppresses virulence and twitching motility	Palanisamy et al., 2010
*Campylobacter jejuni*	Promotes cold growth	Haddad et al., 2009, 2012
*Staphylococcus aureus*	Promotes cold growth	Anderson and Dunman, 2009
*Streptococcus pyogenes*	Degrades virulence transcripts during late exponential phase	Barnett et al., 2007
*Streptococcus mutans*	Is upregulated during acid-shock	Len et al., 2004

*TTSS, Type three secretion system*.

In *S. enterica*, PNPase is required for optimal virulence in both mouse and cell culture infection models (the latter causing acute infections). However, in a seemingly counter-intuitive observation, PNPase works to negatively regulate macrophage invasion and expression of type TTSS genes involved in macrophage invasion (Clements et al., 2002). Additionally, PNPase works to also negatively regulate *Salmonella* plasmid virulence genes (*spv*) transcript levels by acting through the *spv* activator/regulator *spvR*, thereby reducing intracellular survival of *S. enterica* (Ygberg et al., 2006). In *Salmonella*, PNPase might be acting as a molecular switch, whereby a persistent infection is caused unless active PNPase promotes acute infections.

As was the case for the cold shock response, following host immune cell stress, the yersiniae must resume pre-stress gene expression profiles through the removal of unwanted and unnecessary TSSS-associated transcripts. Is PNPase, and, by extension, the degradosome involved in this post-TTSS acclimation following the host-cell encounter. This question becomes even more interesting when considering that both the RNA degradosome and the T3SS are large multi-protein hyperstructure complexes that could be interacting (Norris et al., 2012). Of the 11 known Gram-negative *Yersinia* spp., only *Y. enterocolitica*, *Y. pseudotuberculosis*, and *Y. pestis* are human pathogens. While fecal-oral transmission of *Y. pseudotuberculosis* and *Y. enterocolitica* causes self-limiting gastroenteritis (Galindo et al., 2011), *Y. pestis* (transmitted by the bite of an infected flea) has caused three major human pandemics as well as the great plagues of London in the 1660s (Inglesby et al., 2000). In both *Y. pestis* and *Y*. *pseudotuberculosis*, PNPase was required for optimal virulence in cell culture infections (as measured by bacterial proliferation vis a vis mouse macrophage-like cells and degree of induced HeLa cell rounding, an indirect measure of TTSS function) (Rosenzweig et al., 2005) as well as in murine models of infection (Rosenzweig et al., 2007; Lawal et al., 2011). Interestingly, there was no evidence of PNPase reducing TTSS promoter activity in *Y. pseudotuberculosis* wild type and Δ*pnp* deletion mutants that harbored a *yopE*::*gfp* (Rosenzweig et al., 2005). In contrast, PNPase was required for robust Yop D and E secretion during an early kinetic TTSS induction window (between 0 and 20 min), but not for optimal Yop production levels (Rosenzweig et al., 2005). Counter-intuitively, PNPase promotes robust Yop secretion during initial TTSS induction despite negatively regulating T3SS transcript and protein production levels (Rosenzweig et al., 2007).

Interestingly, different PNPase determinants were required for various yersiniae stress responses (Table 2). Whereas PNPase's catalytic activity was dispensable for inducing HeLa cell rounding, it was absolutely required during cold stress (Rosenzweig et al., 2005). However, it was PNPase's S1 RNA-binding domain that proved necessary for inducing optimal HeLa cell rounding. In fact, S1 RNA-binding domains (derived from exoribonucleases PNPase, RNase II, and RNase R) alone were sufficient in complementing the yersiniae Δ*pnp* deletion mutants when expressed *in trans* (Rosenzweig et al., 2005). Furthermore, the minimal S1 domain region required for Δ*pnp* complementation in TTSS function and HeLa cell cytotoxicity was later determined to be amino acid residues 65–125 (Rosenzweig et al., 2007).

**Table 2:** **List of PNPase determinants in yersiniae stress responses**.

**Organism**	**Degradosome-dependence**	**PNPase role**	**References**
*Yersinia pestis*	?	Catalytic activity plays an essential role in cold growth	Rosenzweig et al., 2005
	?	Required for virulence in a catalytic-activity-independent manner	Rosenzweig et al., 2005
	?	S1 domain sufficient for mediating virulence	Rosenzweig et al., 2005
	?	The first 15 aa residues of the S1 domain are dispensable for virulence; however, the first 30 aa residues are required for virulence	Rosenzweig et al., 2007
	?	Amino acid residue F638 within the PNPase S1 domain is required for virulence and cold growth	Rosenzweig et al., 2007
*Yersinia pseudotuberculosis*	No	S1 and KH domains also contribute to cold growth.	Rosenzweig et al., 2005
	No	Degradosome-independent role in cold growth	Henry et al., 2012
	Yes	Degradosome assembly required for virulence	Yang et al., 2008
	Yes	Degradosome-dependent role in oxidative stress response	Henry et al., 2012
	No	PNPase is dispensable for antibiotic/chemical stress responses	Henry et al., 2012

*S1, RNA-binding domain; KH, RNA-binding domain*.

Within the required 65–125 amino acid S1 domain region, we were able to identify the specific S1 domain residue (F638), which proved necessary for not just TTSS function (as measured by HeLa cell rounding) but also cold growth (Rosenzweig et al., 2007). The yersiniae PNPase F638 is highly conserved and present not just in the S1 domain of *E. coli* PNPase but also in the S1 domain of *E. coli* RNase E at reside F57 (Diwa et al., 2002). Further, a co-crystal structural determination revealed that within the catalytic core of *E. coli*'s RNase E that includes its S1 domain, the F57 residue (conserved with PNPase's F638) might play a facilitating role in binding RNA while residues F67 and K112 actually contact the RNA molecule (Callaghan et al., 2005). Since our previous studies revealed that both the cold-stress response as well as the HCISR require the S1 RNA-binding domain of PNPase (Rosenzweig et al., 2005, 2007; Henry et al., 2012), perhaps an as of yet unidentified upstream regulator (or novel factor) will converge on the PNPase S1 domain's RNA-binding property?

When considering that PNPase confers cold growth capabilities to a number of bacterial pathogens including *Y. pestis*, *Y. enterocolitica, Y. pseudotuberculosis*, *S. enterica*, *C. jejuni*, and *S. aureus* (Goverde et al., 1998; Clements et al., 2002; Rosenzweig et al., 2005, 2007; Anderson and Dunman, 2009; Haddad et al., 2009, 2012), PNPase acts as a “virulence empowering factor” by enhancing the pathogens' survival during adverse/hostile environmental conditions. In another striking example of this, PNPase becomes upregulated ~4-fold in the dental pathogen, *Streptococcus mutans* during acid-shock/encounters with low pH (Len et al., 2004). Perhaps, in the same way PNPase serves as a “virulence empowering factor” during low temperature exposure, it may be enhancing the survival of an oral pathogen when local environments on tooth surfaces become acidic during decay? In another Gram-positive pathogen, PNPase appeared to play a role in *S. pyogenes sag A* (a virulence transcript encoding a streptolysin) and *sda* (encoding an anti-neutrophil DNase) degradation during late-exponential growth phase (Barnett et al., 2007). Taken together, PNPase appears to influence the virulence potential and/or survival of a broad range of pathogens, including the pathogenic yersiniae.

### Do ribonucleases other than PNPase contribute to bacterial stress responses?

PNPase is not the sole ribonuclease involved in bacterial stress responses. As mentioned earlier, RNase R was shown to be up-regulated in *E. coli* as well as being necessary for optimal cold growth (Cairrão et al., 2003). However, in *Y. pestis* neither RNase R nor RNase II was necessary for cold growth in the yersiniae (Lawal et al., 2013). That does not, however, necessarily mean that RNase R and/or RNase II are not also up-regulated in the yersiniae during cold stress, something that has yet to be determined. Interestingly, RNase R has been shown to play a role in the HCISR of *S. flexnari* (Tobe et al., 1992), *Aeromonas hydrophila* (Erova et al., 2008), and *H. pylori* (Tsao et al., 2009). In contrast, RNase R did not appear necessary for *Y. pestis* virulence (Rosenzweig et al., 2005). With regards to the yersiniae, it seems that PNPase specifically influences cold (Rosenzweig et al., 2005, 2007; Henry et al., 2012), oxidative (Henry et al., 2012), and host-cell induced stress responses (Rosenzweig et al., 2005, 2007).

### Degradosome dependence for yersiniae virulence and during oxidative stress but not during cold stress

Using bacterial two-hybrid analysis as well as co-immunoprecipitation techniques, the *Y. pseudotuberculosis* degradosome was determined to contain RNase E, PNPase, and RhlB helicase (Henry et al., 2012), extending an earlier report of physical interaction between only PNPase and RNase E in the aforementioned organism (Yang et al., 2008). To create a degradosome assembly-incompetent *Y. pseudotuberculosis* strain, a dominant-negative, truncated RNase E variant (residues 1–465), containing only its amino terminal catalytic and S1 domains, was ectopically expressed (Briegel et al., 2006; Yang et al., 2008). RNase E autoregulates itself post-transcriptionally; however, when the aforementioned dominant-negative variant was expressed, its autoreagulatory activity was inhibited (Briegel et al., 2006; Yang et al., 2008). Since the carboxy-terminal degradosome scaffolding region was absent when the above mentioned dominant-negative variant was expressed, the yersiniae degradosome could not assemble, as was seen in *Y. pseudotuberculosis* (Yang et al., 2008).

To determine whether degradosome assembly was required for yersiniae virulence, both a proliferation assay in co-culture with macrophage-like cells as well as a TTSS functional evaluation were carried out. Interestingly, *Y. pseudotuberculosis* proliferation was significantly reduced when the degradosome was unable to assemble (Yang et al., 2008). In fact, the degree of reduced proliferation mirrored that of the yersiniae Δ*pnp* deletion mutants observed earlier (Rosenzweig et al., 2005). Furthermore, Yang et al. (2008) were able to demonstrate that degradosome assembly was also necessary for optimum TTSS function, as measured by YopE secretion. Again, this diminished TTSS function resembled that of the yersiniae Δ*pnp* deletion mutants (Rosenzweig et al., 2005, 2007). Taken together, these data collectively demonstrated that the yersiniae requirement for PNPase to achieve optimal virulence is degradosome-dependent.

Was the PNPase contribution to yersiniae cold-growth degradosome-dependent or -independent, and did PNPase contribute to the oxidative stress response? To address this, the dominant-negative, truncated RNase E variant, residues 1–465 (Yang et al., 2008), was again employed. Interestingly, the *Y. pseudotuberculosis* PNPase confers cold-growth capability in a degradosome-independent manner while PNPase responds to oxidative stress, in the form of H_2_O_2_ exposure, in a degradosome-dependent manner (Henry et al., 2012). The yersiniae degradosome dependence for optimal virulence (Yang et al., 2008) does, however, logically align itself with our finding that the *Yersinia* oxidative stress response also requires PNPase in a degradosome-dependent manner (Henry et al., 2012) since, during the host-cell stress, the yersiniae are likely to encounter host-cell generated reactive oxygen species.

In sharp contrast, the PNPase requirement of *E. coli* during oxidative stress was degradosome-independent since an RNase E truncated variant devoid of the degradosome scaffolding region did not demonstrate compromised growth when exposed to H_2_O_2_ (Wu et al., 2009). In *E. coli*, PNPase independently prevented the accumulation of oxidatively damaged RNA (Wu et al., 2009). Curiously, however, PNPase, itself, was required for the optimal oxidative stress response in both *E. coli* and *Y. pseudotuberculosis* (Wu et al., 2009; Henry et al., 2012). Taken together, these data clearly demonstrate that PNPase contributes to various stress responses differently, at times by associating with the degradosome hyperstructure (as in the case of the yersiniae oxidative and host-cell stress response).

## Conclusion

Throughout the literature, many examples of how ribonucleases impact bacterial virulence are apparent (Lawal et al., 2011; Matos et al., 2012; Jester et al., 2012). Whereas RNase R and RNase II have been evaluated for their impacts on bacterial virulence and various stress responses in numerous organisms, detailed studies of the aforementioned ribonucleases in the yersiniae have yet to be carried out. However, PNPase has been well-characterized in the yersiniae and found to be essential for cold growth (Goverde et al., 1998; Rosenzweig et al., 2005), required for optimal T3SS activity (Rosenzweig et al., 2005, 2007), oxidative stress responses (Henry et al., 2012), and yersiniae virulence(Rosenzweig et al., 2005). In fact, the yersiniae are not alone when it comes to dependence on PNPase for optimal virulence. *S. enterica* and *D. nodosus* also require PNPase for optimal virulence, while PNPase works to enhance survival of *C. jejuni*, *S. aureus*, *S. pyogenes*, and *S. mutans* during unfavorable conditions (Table 1). With regards to PNPase's contributions to yersiniae virulence, degradosome assembly was required, indicating that PNPase likely mediates its effect through the larger degradosome hyperstructure. Furthermore, this implicated RNase E, the chief endoribonuclease and scaffold upon which the degradosome assembles, as a virulence-associated factor (Yang et al., 2008).

Despite our furthering the aforementioned study, to determine that yersiniae degradosome assembly was also necessary for oxidative stress responses but not cold stress responses (Henry et al., 2012), still much remains unknown, specifically about the role of other ribonucleases during yersiniae stress responses. Furthermore, the precise mechanism by which PNPase (either in a degradosome-dependent or -independent manner) mediates the specific stress responses mentioned earlier also remains unclear. For example, whether PNPase is degrading a repressor/negative regulator or binding to (through its S1 RNA-binding domain) and protecting an activator/positive regulator remains unknown during the various stress responses. Although progress has been made on understanding the roles the ribonucleases and RNA metabolism during various bacterial stress responses, a broader survey of additional ribonucleases should be carried out in the yersiniae, possibly revealing novel chemotherapeutic targets and/or novel vaccine candidate strains.

### Conflict of interest statement

The authors declare that the research was conducted in the absence of any commercial or financial relationships that could be construed as a potential conflict of interest.
